# Aggressive primary treatments with favourable 5-year survival for screen-interval breast cancers

**DOI:** 10.1186/s12885-018-4319-4

**Published:** 2018-04-06

**Authors:** Gautier Defossez, Alexandre Quillet, Pierre Ingrand

**Affiliations:** 1 Poitou-Charentes General Cancer Registry, Poitiers University Hospital, University of Poitiers, Poitiers, France; 2 INSERM, CIC 1402, Poitiers, France

**Keywords:** Breast neoplasms, Mass screening, Treatment, Survival, Cancer registry, Interval Cancer, Data linkage

## Abstract

**Background:**

To assess the impact of the participation in screening programme according to the mode of detection on the early diagnosis, treatment, and specific survival outcomes in women with breast cancer.

**Methods:**

Women diagnosed with invasive breast cancer in Poitou-Charentes region (France) between 2008 and 2009 were classified into three groups, using data linkage of cancer registry, vital statistics and French organized screening programme: the screening programme (SP), interval cancer (IC), and non-screening programme detected cancer (NSP) groups. Specific survival rates were analysed using the Kaplan–Meier method and Cox proportional hazard models.

**Results:**

Among 1613 patients, 65.7% (*n* = 1059) participated in a screening programme. The interval cancer rate was 17.1% (*n* = 181). Tumours in the IC group were diagnosed at a more advanced stage, i.e. with further regional lymph node metastasis or local spread, than those in the SP group (*p* < 0.001), but with significantly fewer metastases at diagnosis than in the NSP group (p < 0.001). ICs underwent more aggressive primary treatments than the two other groups, with 28% of radical mastectomy and 67% undergoing chemotherapy. The five-year survival rate for IC group were 92.0% (95% CI, 89.9–94.0%).

**Conclusions:**

Interval cancers had more aggressive features than screen-detected cancers but were diagnosed at a less advanced stage compared to non-screen detected cancers. Despite having cancers missed by the screening programme, women who participate in the screening process seem to benefit from early treatment. These results must be confirmed with long-term follow-up.

## Background

Breast cancer is the leading cause of cancer death in women worldwide [1]. Prognosis is mainly determined by the tumour stage at diagnosis [2]. To facilitate early detection and access to effective treatment of breast cancers, screening programmes have been implemented gradually in European countries [3] and have mostly demonstrated the effectiveness of screening in reducing breast cancer mortality [4–9].

However, the benefit of mammogram screening is still regularly challenged by controversies, regarding overdiagnosis, false-positive results, possibly radiation-induced cancer or interval cancers [10–12]. Moreover, no reduction in breast cancer mortality was observed in a Canadian randomized mammography screening trial [13]. These controversies add complexity to informed decision making for clinicians and patients, and create negative feedback for screening programmes.

In France, a screening programme was implemented nationwide since 2004, and offers a physical examination and a bilateral mammogram biennially to women aged 50–74 years. Ten years after its establishment, only slightly more than half of women (52.1% in 2014) participate, and 10% of women choose an individual (opportunistic) screening with a mammography performed under medical prescription, outside the official programme [14, 15].

Further investigations are needed to deliver an objective and comprehensible message to women and policy makers. The special case of interval cancers that have a potential influence on the effectiveness of screening should be considered. Interval cancers, although they are considered as false-negatives of the screening programme, usually become clinically evident shortly after the last normal screening result. Interval cancers often reflect aggressive tumours encountered in women already involved in the screening programme [16–18]. This fast-growing lump in the breast may be readily detected by self-examination and would also give rise to more anxiety than a slow-growing one, leading to clinical examination [19]. Whereas their clinical and biological characteristics are now better documented, inconsistent findings exist on their prognosis in the literature and no study to our knowledge has taken into account treatments as an indicator of screening programme effectiveness [20–25].

This study aimed to assess the impact of the participation in screening programme according to the mode of detection on the early diagnosis, treatment, and specific survival outcomes in women with breast cancer, using data linkage of cancer registry, vital statistics and French screening programme.

## Methods

### Patients

Women aged 50–74 years, residing in the Poitou-Charentes region (1.8 million inhabitants, South-West France) with the first diagnosis of invasive breast carcinoma between 1 January 2008, and 31 December 2009, were included in this study.

### The French screening programme

In France, the breast cancer screening programme is offered biennially to women aged 50–74 years. It includes a physical examination and a bilateral mammogram, the results of which are based on the Breast Imaging-Reporting And Data System (BI-RADS) classification of the American College of Radiology. The BI-RADS 1 (negative) and 2 (benign finding) mammograms are systematically subjected to a second reading aimed to reduce the false-negative rate. For other patients as BI-RADS 0, 3, 4 or 5, follow-up or complementary diagnostic procedure (biopsy, ultrasonography, magnetic resonance imaging) with or without a specific mammographic follow-up are provided. The screening programme and data registration are conducted by the screening facilities located in each of the four French administrative departments (counties) of the Poitou-Charentes region. Screening mammograms performed outside the invitation of the screening programme (individual screening) are not included.

### Data

Primary invasive breast carcinomas were identified from the Poitou-Charentes cancer registry between 1 January 2008, and 31 December 2009. For each case, patient, tumour, and healthcare data were routinely reported according to international rules [26]. In the present study, the prognostic variables included age, tumour stage classified according to the TNM classification of malignant tumours, histological Scarff-Bloom-Richardson grade, oestrogen and progesterone receptor status, and human epidermal growth factor receptor 2 (HER-2) expression. Cancer treatments (neoadjuvant treatment, surgery, adjuvant chemotherapy, and radiotherapy) were also recorded [27, 28]. Hormone therapy was not reported outside neo-adjuvant hormone therapy.

The dates and results of screening programme mammograms were obtained from the four screening facility databases of Poitou-Charentes. Patients were classified according to the mode of detection as: Screen-detected cancers from the Screening Programme (SP group) were defined as women having a positive mammography (BI-RADS 3, 4, or 5) followed by complementary diagnostic procedures including histological confirmation of cancer. The interval cancer group (IC group) were defined as women having a negative mammography (BI-RADS 1 or 2) followed by a histological diagnosis of cancer occurring within the 24 months of the prior mammogram. The cut-off of 24 months corresponded to the waiting time, recommended in the French screening programme, between two screening mammograms. The non screen-detected cancer group (NSP group) were defined as women having a histological diagnosis of cancer without having participated in screening programme and could include opportunistic screening or breast cancers detected based on clinical signs or symptoms. A BI-RADS 0 (incomplete assessment) mammogram was a temporary classification that required complementary diagnostic action (extension, ultrasonography, biopsy). In the absence of reclassification for BI-RADS 1 or 2, these mammograms were considered positive and included in the SP group.

Patients’ vital information until 31 December 2014 was obtained from data of the French national civil registration file RNIPP, maintained by the National Institute of Statistics and Economics Studies. French native patients who had not been reported dead were censored at December 31, 2014. Foreign patients were censored at the date of the last follow-up. A systematic review of the medical records was performed to identify the cause of death. Death was related to breast cancer in the presence of disease progression.

This study was approved by the French regulatory authorities (the “Comité Consultatif sur le Traitement de l’Information en matière de Recherche dans le Domaine de la Santé” and the “Commission Nationale Informatique et Libertés”, authorisation number 907303). According to French law, patients were informed of their data registration and given the right to deny access or to rectify their personal data.

### Statistical analyses

Patient, tumour, and healthcare characteristics of IC were compared to the two other modes of detection using the Chi-squared or Fisher’s exact test. Survival rates were estimated using the Kaplan–Meier method. Hazard ratios and their 95% confidence intervals were estimated from univariate and multivariate Cox proportional hazard models. For specific survival analysis, women who died of non-breast cancer-related causes were censored at the date of death. Searching for interactions and collinearity between included variables was performed in the multivariate analysis. Two multivariate analysis were performed, with and without adjustment on main prognostic factors, in order to highlight the important role of TNM stage at diagnosis in the interpretation of the results. The threshold *p*-value for including variables in the final multivariate Cox model was set at 5% except for the detection mode variable, which was forced into this model. Data management and statistical analyses were performed using SAS software version 9.4 (SAS Institute Inc., Cary, NC, USA).

## Results

**Fig. 1:**
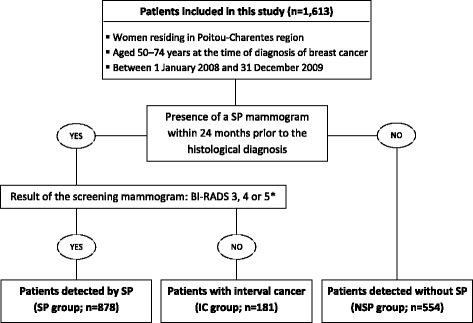
Inclusion of patients according to the mode of detection. * According to the Breast Imaging-Reporting And Data System (BI-RADS) classification of the American College of Radiology

This registry-based study included 1613 patients aged 50–74 years at breast cancer diagnosis. Among these patients, 1059 (65.7%) underwent a screening programme mammogram within 24 months before diagnosis, which revealed tumours in 878 (82.9%) patients (Fig. 1). Therefore, the interval cancer rate was 17.1% (*n* = 181).

### Comparison of prognostic and treatment characteristics

The distribution of characteristic and prognostic factors according to the mode of detection is shown in Table 1. Tumours in the IC group were diagnosed at a more advanced stage (*p* < 0.001), with higher-grade tumours (*p* < 0.001), and with a greater proportion of hormone receptor negative tumours (*p* < 0.001) compared with those in the SP group. Conversely, tumours in the IC group were diagnosed at a significantly less advanced stage (*p* < 0.001) compared with those in the NSP group. Distant metastasis were reported in 11.7% of the NSP group vs 1.0% in the SP group and 3.9% in the IC group.

**Table 1:** Distribution of patient, tumour, and treatment characteristics of invasive breast cancer according to the mode of detection

	SP group (*n* = 878)		IC group (*n* = 181)		NSP group (*n* = 554)		SP vs. IC group	SP vs. NSP group	IC vs. NSP group
	n	(%)	n	(%)	n	(%)	p	p	p
Age							0.060	0.307	0.236
> 65 years	319	(36.3)	52	(28.7)	186	(33.6)			
≤ 65 years	559	(63.7)	129	(71.3)	368	(66.4)			
TNM stage							< 0.001	< 0.001	< 0.001
I	560	(63.8)	67	(37.0)	227	(41.0)			
II	255	(29.0)	86	(47.5)	174	(31.4)			
III	47	(5.4)	21	(11.6)	77	(13.9)			
IV	9	(1.0)	7	(3.9)	65	(11.7)			
Unknown	7	(0.8)	0	(0.0)	11	(2.0)			
Extent of disease							< 0.001	< 0.001	0.0014
Tumor with local spread (any T/N0/M0)	647	(73.7)	105	(58.0)	315	(56.9)			
T1	560	(86.6)	67	(63.8)	227	(72.1)			
T2	78	(12.1)	31	(29.5)	71	(22.5)			
T3	8	(1.2)	5	(4.8)	5	(1.6)			
T4	1	(0.1)	2	(1.9)	12	(3.8)			
Tumor with regional spread (any T/N+/M0)	215	(24.5)	69	(38.1)	163	(29.4)			
Advanced cancer (any T/any N/M+)	9	(1.0)	7	(3.9)	65	(11.7)			
Unknown	7	(0.8)	0	(0.0)	11	(2.0)			
SBR grade							0.001	0.022	0.147
1	228	(26.0)	27	(14.9)	115	(20.8)			
2	486	(55.4)	109	(60.2)	299	(54.0)			
3	136	(15.5)	42	(23.2)	110	(19.9)			
Unknown or not assessed	28	(3.2)	3	(1.7)	30	(5.4)			
Hormonal receptor status							< 0.001	< 0.001	0.171
OR+/PR+	633	(72.1)	105	(58.0)	331	(59.8)			
OR+/PR- or OR-/PR+	137	(15.6)	32	(17.7)	109	(19.7)			
OR-/PR-	88	(10.0)	41	(22.7)	89	(16.1)			
Unknown or not assessed	20	(2.3)	3	(1.7)	25	(4.5)			
Her-2 receptor status							0.350	0.068	0.899
Positive	86	(9.8)	23	(12.7)	72	(13.0)			
Negative	699	(79.6)	146	(80.7)	427	(77.1)			
Unknown or not assessed	93	(10.6)	12	(6.6)	55	(9.9)			
Type of treatment							< 0.001	< 0.001	< 0.001
Surgery ± RT	535	(60.9)	60	(33.2)	233	(42.1)			
Surgery + CT ± RT	293	(33.4)	91	(50.3)	199	(35.9)			
Neoadjuvant treatment ^a^+ Surgery ± CT/RT	43	(4.9)	27	(14.9)	68	(12.3)			
No surgery (refusal, palliative treatment)	3	(0.3)	3	(1.7)	45	(8.1)			
Unknown	4	(0.5)	–	–	9	(1.6)			
Type of surgery							0.045	< 0.001	0.113
Breast-conserving surgery	692	(78.8)	129	(71.3)	327	(59.0)			
Mastectomy	178	(20.3)	49	(27.1)	171	(30.9)			
Unknown or no surgery	8	(0.9)	3	(1.7)	56	(10.1)			

Chi-square and Fisher’s exact test did not including any missing values

SP group, patients detected by the screening programme; IC group, patients with interval cancer; NSP group, patients detected without participating in the screening programme; SBR, Scarff-Bloom-Richardson grade; OR, oestrogen receptor; PR, progesterone receptor; RT, adjuvant radiotherapy; CT, adjuvant chemotherapy

^a^Neoadjuvant treatment include neoadjuvant chemotherapy (38/43 in SP group, 25/27 in IC group and 57/68 in NSP group) or neoadjuvant hormonotherapy (5/43 in SP group, 2/27 in IC group and 11/68 in NSP group). One patient in NSP group receive both neoadjuvant hormonotherapy and adjuvant chemotherapy

The proportion of regional lymph node metastasis were significantly higher for non-metastatic cancers in the IC group than in the SP group (38.1% vs. 24.5%, *p* < 0.001). For tumours without lymph node involvement, 36.3% of tumours in the IC group were larger than 2 cm (T2-T3-T4) vs. 13.4% in the SP group.

Concerning treatment, breast conserving surgery was less frequently performed in the IC group compared with the SP group, with a higher proportion of mastectomy (28% of mastectomy in the IC group vs. 20% in the SP group, *p* = 0.045). Chemotherapy (adjuvant or neoadjuvant) was more frequently performed in the IC group compared with the two other groups (38% in SP group, 64% in IC group and 46% in NSP group, *p* < 0.001). Patients in the IC group underwent significantly less palliative care (*p* < 0.001) compared with the NSP group.

### Comparison of survival

**Fig. 2:**
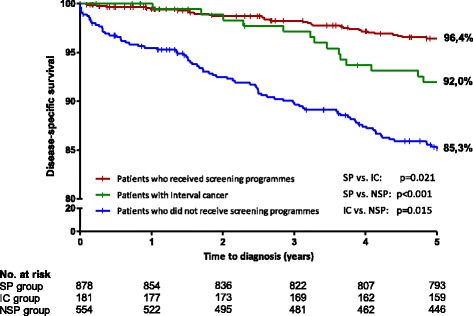
Disease-specific survival probability of patients with invasive breast cancer according to the mode of detection

The median follow-up for the study group was equal to 5.8 years (interquartile range, 5.3–6.4 years). One hundred and eighty-eight (11.7%) women died during the follow-up period, including 136 (72.3%) breast-cancer-related deaths. Kaplan-Meier survival curves are shown in Fig. 2. The 5-year specific survival rate was 92.0% in the IC group (95% CI, 89.9–94.0%), 96.4% in the SP group (95% CI, 95.8–97.1%) and 85.3% in the NSP (95% CI, 83.8–86.9%). Superior survival was observed in the IC group compared with the NSP group (*p* = 0.015). After 3 years, a significant survival difference emerged between the SP and IC groups (*p* = 0.021).

Univariate and multivariate Cox regression analyses are shown in Table 2. Due to collinearity between the treatment type and prognostic factors, treatment type was not included in the multivariate analysis. The final model retained three independent prognostic factors including an early stage at diagnosis (*p* < 0.001), hormone receptor-positive tumours (*p* < 0.001), and age ≤ 65 years (*p* = 0.003). Detection mode was not significant in survival analysis when taking into account TNM stage at diagnosis. A significant survival was observed in the IC group compared to the NSP group in the unadjusted model (*p* < 0.001). Analyses of overall survival confirmed these findings, except for the difference between the SP and IC groups, which was no longer significant (*p* = 0.40), with the 5-year overall survival rates being 93.7% in the SP group, 90.9% in the IC group, and 82.4% in the NSP group.

**Table 2:** Hazard ratios (HR) and 95% confidence intervals (CI) of prognostic factors in patients with invasive breast cancer by univariate and multivariate Cox regression analysis

	Univariate analysis			Multivariate analysis					
				With adjustment on TNM stage			Without adjustment on TNM stage		
	HR	95% CI	p	HR	95% CI	p	HR	95% CI	p
Mode of detection			< 0.001			0.325			< 0.001
NSP group	1			1			1		
SP group	0.25	0.17–0.37		0.75	0.48–1.15		0.28	0.19–0.42	
IC group	0.51	0.29–0.87		0.74	0.42–1.30		0.48	0.28–0.83	
Age			0.128			0.003			0.05
> 65 years	1			1			1		
≤ 65 years	0.77	0.54–1.08		0.58	0.41–0.84		0.70	0.49–0.99	
TNM stage			< 0.001			< 0.001			–
I	1			1			–		
II	2.62	1.40–4.90		2.59	1.33–5.02		–	–	
III	14.48	8.03–26.09		13.25	7.02–25.01		–	–	
IV	76.92	44.12–134.08		63.69	33.69–120.39		–	–	
SBR grade			< 0.001			–			–
1	1			–			–		
2	3.02	1.56–5.84		–	–		–	–	
3	5.82	2.93–11.59		–	–		–	–	
Hormonal receptor status			< 0.001			< 0.001			< 0.001
OR+/PR+	1			1			1		
OR+/PR- or OR-/PR+	2.78	1.82–4.25		1.97	1.28–3.02		2.49	1.63–3.81	
OR-/PR-	4.30	2.87–6.45		2.88	1.89–4.38		3.89	2.58–5.87	
Her-2 receptor status			0.050			–			–
Positive	1			–			–		
Negative	0.63	0.40–1.00		–	–		–	–	
Type of treatment			< 0.001			–			–
Surgery ± RT	1			–			–		
Surgery + CT ± RT	3.75	2.18–6.45		–	–		–	–	
Neoadjuvant treatment + Surgery ± RT/CT	11.36	6.33–20.39		–	–		–	–	
Absence of surgery	84.72	48.26–148.73		–	–		–	–	

SP group, patients detected by the screening programme; IC group, patients with interval cancer; NSP group, patients detected without participating in the screening programme; SBR, Scarff-Bloom-Richardson grade; OR, oestrogen receptor; PR, progesterone receptor; RT, adjuvant radiotherapy; CT, adjuvant chemotherapy

## Discussion

Interval cancers are, as suggested by our study, diagnosed at a significantly less advanced stage compared with those in the NSP group. They show more aggressive features than screen-detected cancers, while undergoing more aggressive primary treatments with a higher rate of mastectomy and two-thirds undergoing chemotherapy. The individual data available for each patient confirm the linear gradient of TNM stage according to the mode of detection and provides interesting additional information on the initiated treatment. Most tumours in the SP group are localized cancers classically treated by breast-conserving surgery and radiotherapy. Tumours in the IC group are characterized by more local and regional spread justifying a more aggressive treatment with neoadjuvant therapy or adjuvant chemotherapy. Tumours in the NSP group show significantly more advanced cancers with metastastic and non-resectable cancers characterized by a greater proportion of palliative care.

As a majority of studies, we have found no significant differences in prognosis between women with interval cancer compared with an unscreened population, when taking into account the main prognostic factors [20–25]. The IC group had superior 5-year survival rates compared with the NSP group in univariate model, but this difference became reasonably non-significant after adjustment because of the strong survival advantage attributed to differences in the initial distribution of TNM stage at diagnosis [29–31]. The multivariate Cox model without adjustment on TNM stage confirmed the better and significant survival in the IC group than in the NSP group. Supplementary individual information on diagnosis and treatment provides essential results to properly understand the benefit on breast cancer survival.

We assume that the appropriate method for determining whether a cancer screening strategy works is the randomized controlled trial, with mortality as the endpoint. But the study of interval cancer on case diagnosis, treatment and survival is interesting, while the emphasis is now on evaluation of routine screening services for which randomized trials may not be suitable [31]. The cancer registry provides robust data, ensuring the completeness of incident cancer cases and thereby avoiding selection bias. The use of individual data allowed the monitoring of a screening programme in a real setting, and with controlling for individual differences that might affect the primary outcome, particularly TNM stage at diagnosis [2]. Studies usually covered a period where the national screening programme was implemented gradually or referred to old data. Our study was initiated 4 years after the generalisation of the screening programme, which placed the analysis in a stable situation regarding diagnostic procedures, participation and care in breast cancer.

This study has some limitations. The present study did not allow to assess the occurrence of mammograms performed outside the screening programme (opportunistic screening) [32]. In a report issued by the French National Authority for Health [14], the participation for this mode of detection was estimated at 10% of the target population. The authors highlighted the difficulties in identifying these patients and the heterogeneity of practices regarding this type of screening. If individually screened patients could be distinguished from non-screen detected patients, the 5-year survival rate might have been even worse, and survival difference between non-screen detected patients and the others might have been increased. Second, misclassification of the mode of detection cannot be excluded. Some interval cancers could be classified as screen-detected cancers if symptomatic women waited for the screening mammography instead of a consultation with the physician. Third, we did not interpret the results concerning survival differences between the SP group and the two others because of known biases such as lead-time and length-time which invariably provide a higher survival in screen-detected cancers.

## Conclusions

In conclusion, more aggressive treatments were found in patients with interval cancers. Despite the aggressiveness of these cancers, women who participate in the screening process seem to benefit from early treatment. These results must be confirmed with long-term follow-up. Such a result could not be explained by overdiagnosis, but instead appeared to be the consequence of a reduction in late diagnosis due to participation in screening programme and an access to suitable and curative treatments. These findings reinforce the need to promote organised screening. Participation in a screening programme was important in facilitating early detection in these women. A survival benefit might be expected by increasing the participation rate in screening programmes if they are accessible to everyone at risk.

## Abbreviations

BI-RADS: Breast Imaging-Reporting and Data System
CT: Adjuvant chemotherapy
HER-2: Human Epidermal Growth Factor Receptor 2
IC: Interval Cancer
NSP: Non-Screening Programme
OR: Oestrogen Receptor
PR: Progesterone Receptor
RT: Adjuvant Radiotherapy
SBR: Scarff-Bloom-Richardson grade
SP: Screening Programme
